# Value for Money

**Published:** 1920-01-31

**Authors:** 


### VALUE FOR MONEY.

THE services of the medical profession in connection with State departments and the general question of the efficiency of Insurance Act administration have been given much prominence by events of the last week or two. It is obvious there is much that is unsatisfactory, and the medical profession has shown commendable determination to use its efforts towards bringing about such reorganisation as will give the community value for its money and the medical men an unhampered opportunity of rendering their best services. They assert that neither of these conditions at present exists, and the Federation of Medical and Allied Societies has pressed the Minister of Health for an immediate public inquiry, to which the Minister has given a sympathetic, but indefinite, reply.

The position of the great majority of medical men is described in a statement of much public interest published in the *Sunday Times*. The General Secretary of the Federation there says that the Insurance Act has been in force for seven years without any investigation as to its effectiveness. It is doubtful whether the insured public can be satisfied with the existing services, and it is certain that the panel system never has brought, and never can bring, to the bedside of the insured person the most modern methods of treatment and investigation. This is a serious statement, coming, as it does, from a responsible medical man representing a large proportion of his colleagues throughout the country, and it is calculated to stimulate public opinion to back the demand for an immediate inquiry.

Dr. Addison is a faithful henchman of the author of the Insurance Act, and may not like too frank a disclosure of the workings of the measure, but when we have the warning that the example of Kent practitioners in resigning all connection with panel practice may be followed elsewhere, no political considerations must be allowed to prevent an honest endeavour to remedy this serious state of affairs.

The Federation hopes to have an opportunity of placing

THE PUBLIC WELL-BEING—(*continued*).

its views before the Prime Minister. If the doctors declare that the rules and regulations prevent them from giving their patients efficient service, the public—which pays for this inefficient care of its health—has every right to demand an explanation and, if necessary, reform. What objection can there be, on common-sense grounds, to the immediate inquiry asked for? Everybody knows the Insurance Act, on the financial side alone, has been little short of a scandal, and if all this extravagance has brought only "a few words and bottles of cheap medicine" in place of treatment, the public ought to know why.

